# Whole-Genome Shotgun Sequencing of *Rhodococcus erythropolis* Strain P27, a Highly Radiation-Resistant Actinomycete from Antarctica

**DOI:** 10.1128/genomeA.00763-13

**Published:** 2013-09-26

**Authors:** Rodrigo Gouvêa Taketani, Tiago Domingues Zucchi, Itamar Soares de Melo, Rodrigo Mendes

**Affiliations:** Laboratory of Environmental Microbiology, EMBRAPA Environment, Jaguariúna, São Paulo, Brazil

## Abstract

Here, we report the draft genome sequence of radiation-resistant *Rhodococcus erythropolis* strain P27, isolated from leaves of *Deschampsia antarctica* Desv. (*Poaceae*) in the Admiralty Bay area, Antarctica.

## GENOME ANNOUNCEMENT

It is becoming increasingly clear that global warming influences the climate and affects living beings in many ways (1). This global phenomenon also affects the ozone layer (2). The resulting depletion of the ozone layer usually leads to deleterious effects, mainly due to high UV incidence, which may pose a special threat for organisms living in Antarctica. In our survey of the epiphytic bacteria associated with *Deschampsia antarctica* Desv. (*Poaceae*), one of only two native flowering plants occurring throughout maritime Antarctica, we recovered an actinomycete isolate moderately to highly resistant to gamma rays and UVC radiation.

Members of the *Rhodococcus* genus have been intensively studied owing to their ability to survive under extreme conditions (3, 4), and these bacteria seem to be interesting targets for screening radiation-resistant genes (5). Furthermore, although many investigations have demonstrated the resistance of *Rhodococcus* bacteria to UVC exposure, there is a lack of information on their resistance to gamma radiation. To gain insight into the genes related to the resistance to ionizing and nonionizing radiation of strain P27, we performed whole-genome sequencing using the Ion Torrent (PGM) platform.

Sequencing was carried out on the Ion 316 chip provided in the Ion sequencing kit 200 v.2.0, following the manufacturer’s protocol. The genome sequence was *de novo* assembled using the MIRA v.3.4, CLC Genomics Workbench v.5.5.1, and SeqMan NGen v.4.0.0 packages, and the obtained contigs were integrated by using CISA (6). The taxonomic position of strain P27 was further evaluated by using the JSpecies package (7).

The total number of reads (>Q20) generated using *R. erythropolis* PR4 (GenBank accession no. AP008957.1) as a reference was 2,972,204, which were allocated into 60 contigs with 85.3× coverage and a mean length of 123.25 bp. The assembled data were analyzed by RAST annotation (8), and the genome size was found to be 6,262,348 bp, comprising 6,852 open reading frames (ORFs). The G+C content was estimated to be 62.4 mol%. The genome contains 78 copies of 12 genes annotated as DNA repair mechanisms usually associated with radiation resistance, mainly DNA repair base excision and DNA repair bacterial *recBCD* and *recFOR* pathways. 16S rRNA gene analysis revealed that strain P27 (CMAA 1247) shares high identity with the type strain of *Rhodococcus erythropolis* (9). The average nucleotide identity (ANI) of P27 and its phylogenetically closely related neighbor was 98.5%, suggesting that P27 is a member of the *R. erythropolis* species.

### Nucleotide sequence accession numbers.

The *R. erythropolis* strain P27 genome sequence and annotation data have been deposited at DDBJ/EMBL/GenBank under the accession number AVCO00000000. The version described in this paper is version AVCO01000000.
